# Necrotizing Fasciitis Associated with *Staphylococcus lugdunensis*

**DOI:** 10.1155/2012/453685

**Published:** 2012-05-17

**Authors:** Tony Hung, Soroush Zaghi, Jonathan Yousefzadeh, Matthew Leibowitz

**Affiliations:** ^1^Department of General Surgery, David Geffen School of Medicine at UCLA, Los Angeles, CA 90095, USA; ^2^Division of Infectious Diseases, Department of Medicine, David Geffen School of Medicine at UCLA, Los Angeles, CA 90095, USA

## Abstract

Necrotizing fasciitis is a life-threatening soft tissue infection that results in rapid local tissue destruction. Type 1 necrotizing fasciitis is characterized by polymicrobial, synergistic infections that are caused by non-Group A *streptococci*, aerobic and anaerobic organisms. Type 2 necrotizing fasciitis involves Group A *Streptococcus* (GAS) with or without a coexisting staphylococcal infection. Here we provide the first report of necrotizing fasciitis jointly associated with the microbes Group B *Streptococcus* and *Staphylococcus lugdunensis*. *S. lugdunensis* is a commensal human skin bacterium known to cause often painful and prolonged skin and soft tissue infections. To our knowledge, however, this is the first case of *Staph. lugdunensis*-associated necrotizing fasciitis to be reported in the literature.

## 1. Introduction

 The spectrum of disease-causing microbes continues to change rapidly. Not only are we documenting an alarming increase in antimicrobial resistance but also we are now detecting an emerging tendency for infectious disease pathogens to develop greater degrees of virulence. Necrotizing fasciitis (NF) is a rapidly progressing, life- and limb-threatening soft tissue infection that targets the superficial fascial tissue layer. The disease is characterized by quickly spreading erythema, pronounced tenderness, severe pain, subcutaneous gas, fever, and tachycardia [1]. Whereas a number of pathogens including Group A* Streptococcus*, Enterobacteriaceae, *Clostridium perfringens*, and *Staphylococcus aureus* have commonly been associated with necrotizing fasciitis, a growing number of unprecedented bacteria are also becoming associated with this severe soft tissue infection. As necrotizing fasciitis is a potentially lethal and devastating infectious disease process, it is important that novel virulence patterns be documented and addressed as soon as they are detected. Here we report the first case of necrotizing fasciitis to be associated with the microbe *Staphylococcus lugdunensis*.

## 2. Case

 A 66-year-old African American woman with osteoporosis, osteoarthritis, and impaired glucose tolerance presented to the emergency department with a 5-day history of left groin pain, nausea, vomiting, and fatigue. Five days prior to admission, she had noted an abscess on her inner left thigh that progressively ruptured and drained purulent, bloody material. On evaluation, she was tachycardic (HR: 111–136) and febrile to 103.2°F. The left thigh wound drained a thin serous and dishwater-type fluid. Labs revealed leukocytosis and hyperglycemia (WBCs = 23.8 × 10^3^: 86.4% neutrophils, 5.4% lymph, 8.1% monocyte; glucose: 239 g/dL; BUN 13 mg/dL, creatinine 0.6 mg/dL, chloride 92 mmol/L, total CO_2_ 24 mmol/dL, total creatinine kinase = 67 mg/dL). CT scan showed a 4.6 × 1.4 × 5 cm fluid collection proximal to the left gracilis muscles with presence of gas bubbles, edema, and fat stranding in the subcutaneous tissues. The patient was taken emergently to the operating room to undergo radical debridement, washout, packing, and subsequently admitted for IV antibiotic therapy.

**Figure 1:**
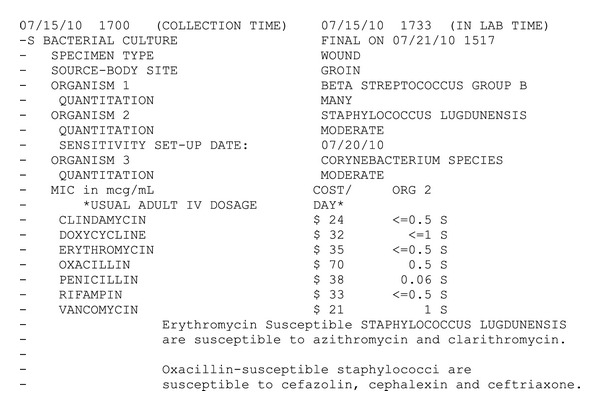
Microbiology culture and sensitivity report from initial debridement.

 Microscopic analysis of the debrided specimen revealed areas of necrosis and acute and chronic inflammation, consistent with a diagnosis of necrotizing fasciitis. Gram stain of the initial intraoperative specimen showed “*few gram positive cocci in pairs and chains,*” and final bacterial culture showed many GBS, moderate *S. lugdunensis*, and moderate *Corynebacterium* (see Figure 1). Subsequent additional debridement grew many GBS and many *S. lugdunensis*. Blood cultures were negative. Blood tests for HIV 1, HIV 2, hepatitis B, and hepatitis C were negative. QuantiFERON gold tuberculosis test was negative. During the course of hospitalization, the patient was empirically treated with IV antibiotics including vancomycin, clindamycin, and aztreonam. After multiple debridements, the patient underwent plastic surgery reconstruction of her left groin. She was discharged home in stable condition after a 67-day hospital course and is doing well at followup 18 months later.

## 3. Discussion

 Necrotizing fasciitis (NF) is a life-threatening soft tissue infection that results in rapid local tissue destruction of primarily the superficial fascia; the infective process is characterized by angiothrombotic microbial invasion and liquefactive necrosis of the deep dermis and fascia [1]. Estimated 500 to 1,500 cases of NF are diagnosed each year in the United States with a case-fatality rate of 24% [2]. While surgical debridement is the essential treatment of NF, addition of broad-spectrum antibiotics at the time of presentation and further appropriate antimicrobial therapy targeted for specific causative microbes may modify the clinical picture and are also critical in the management of this infectious process [1, 2].

 NF is traditionally classified into two distinct categories based on the causative microorganisms [2]. Type 1 NF is characterized by polymicrobial, synergistic infections that are caused by non-Group-A streptococci, aerobic and anaerobic organisms [1]. Type 2 NF involves Group A* Streptococcus* (GAS) with or without a coexisting staphylococcal infection [2]. While the clinical manifestations are similar, type 1 and type 2 NF tend to affect different subgroups of patients. Type 1 NF has a propensity to affect diabetic and immunocompromised individuals, whereas type 2 NF tends to occur in individuals with no underlying comorbidities [1]. Both type 1 and type 2 NF are considered surgical emergencies; appropriate management requires comprehensive surgical debridement combined with targeted antimicrobial therapy and physiologic support [3].

 Because of the importance of targeted antibiotic therapy in the treatment of NF, awareness of the spectrum of microbes that might be associated with NF is imperative. Microbes commonly associated with NF include: Group A* Streptococcus*, *Clostridium perfringens*, methicillin-sensitive *Staphylococcus aureus*, Enterobacteriaceae*, E. coli, Pseudomonas *spp.*, Klebsiella *spp.*, V. vulnificus, A. hydrophila, *and anaerobic *Streptococcus* spp. [1]. While these microbes comprise most of all cases, other pathogens such as MRSA and Group B* Streptococcus* (GBS) have also been associated with NF on rare occasions [4]. Here we document for the first time a case of NF caused by a polymicrobial combination of Group B* Streptococcus* and *Staphylococcus lugdunensis*.

*Staphylococcus lugdunensis* is a commensal human skin bacterium first described in 1988 and since reported as a cause of prolonged and recurrent skin and soft tissue infections, as well as serious infections including meningitis, ventriculoperitoneal shunt infection, spondylodiscitis, prosthetic joint infection, catheter-related bacteremia, endocarditis, and pacemaker-related infections [5, 6]. *S. lugdunensis* is a coagulase-negative Staphylococcus with a pathogenicity and virulence more similar to *Staphylococcus aureus* than to other coagulase-negative *Staphylococcus* spp.. In fact, these organisms are frequently misidentified as *S. aureus* because of their morphologic appearance with yellow pigmentation and complete hemolysis when cultured on blood agar. Even so, appropriate identification of *S. lugdunensis* is imperative as misidentification may result in inadequately treated, prolonged, and persistent infection [6].

 Indeed, *S. lugdunensis* comprises a niche that is distinctly different from those of *S. aureus* [7, 8]; *S. lugdunensis* is an integral part of the normal skin flora, predominant at the abdomen, groin, and lower extremities (especially the nail bed of the first toe) and is only rarely obtained from cultures of nasal swabs—a pattern of distribution distinct from *S. aureus* [7, 9]. Moreover, its characteristic antibiotic susceptibility profile is unusual for both coagulase-negative and coagulase-positive staphylococci. *S. lugdunensis* typically lacks the mecA gene, which codes for penicillin binding protein (PBP) 2a and induces methicillin resistance [10]. Moreover, because only about 20% of isolates are beta-lactamase-positive, it is often possible to use penicillin for treatment of infections caused by these organisms. In addition, *S. lugdunensis* is also usually susceptible to gentamicin, rifampicin, vancomycin, oxacillin, and cefoxitin, and <10% of isolates are found to be resistant to clindamycin or fusidic acid [5].

 According to a 5-year consecutive record review by Herchline and Ayers, approximately 10.1% of isolated staphylococcal species that were not *S. aureus* or *S. epidermidis* were indeed identified as *S. lugdunensis *[11]. The most common clinical diagnoses were skin and skin structure infections (55.4%) and blood and vascular catheter infections (17.4%). For 40% of the reviewed specimens, *S. lugdunensis* was the sole agent isolated, and for 60% of specimens, *S. lugdunensis* was isolated as part of mixed flora. In only 15.4% of clinically reviewed specimens was *S. lugdunensis *clearly a culture contaminant or colonizing organism. The pattern of human infection identified in that study emphasizes the predominance of *S. lugdunensis* to affect skin and soft tissue structures [11]. Even so, this here is the first report of an association of *S. lugdunensis *with the fulminant manifestation of necrotizing fasciitis.

## 4. Conclusion

*S. lugdunensis* is a common cause of skin and softtissue infections (SSTIs) that is likely underrated by many laboratories. *S. lugdunensis* is distinct in both niche, clinical spectrum of disease, and antibiotic sensitivity in comparison to other staphylococcal species. This case report demonstrates the potential of *S. lugdunensis* to cause SSTI as extensive as necrotizing fasciitis. As such, *S. lugdunensis *should be accepted as a clinically significant human pathogen and should be distinctly identified and included in all routine bacteriological examinations. Clinicians should be acquainted with the name and the pathology of the bacterium. Moreover, this case report also identifies Group B* Streptococcus* as a synergistic microbe in this case of NF associated with *S. lugdunensis*.
